# Supplementary material for: Dealing with uncertainty in agent-based models for short-term predictions
Source: R Soc Open Sci. 2020 Jan 15;7(1):191074. doi: 10.1098/rsos.191074 (PMC7029931; doi:10.1098/rsos.191074)
Supplement: Supplementary material [file rsos191074supp1.zip › Figures/framework_rev.pdf]

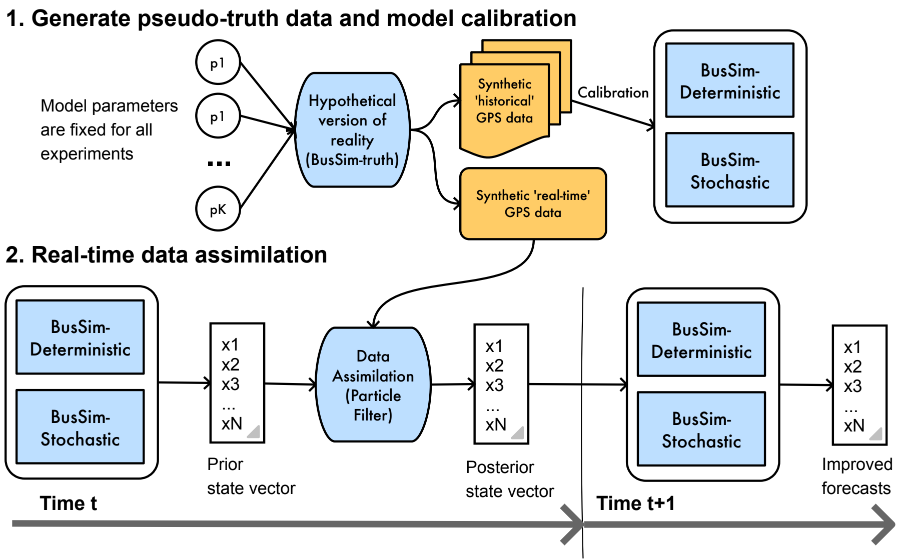
1. Generate pseudo-truth data and model calibration
Model parameters are fixed for all experiments
p1
p1
...
pK
Hypothetical version of reality (BusSim-truth)
Synthetic 'historical' GPS data
Calibration
BusSim-Deterministic
BusSim-Stochastic
Synthetic 'real-time' GPS data
2. Real-time data assimilation
BusSim-Deterministic
BusSim-Stochastic
x1
x2
x3
...
xN
Prior state vector
Data Assimilation (Particle Filter)
x1
x2
x3
...
xN
Posterior state vector
BusSim-Deterministic
BusSim-Stochastic
x1
x2
x3
...
xN
Improved forecasts
Time t
Time t+1
