# Supplementary figures and images for: Dealing with uncertainty in agent-based models for short-term predictions
Source: R Soc Open Sci. 2020 Jan 15;7(1):191074. doi: 10.1098/rsos.191074 (PMC7029931; doi:10.1098/rsos.191074)

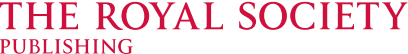

Supplement: Supplementary material [file rsos191074supp1.zip › RSOS_Pubs_Logo_Line_CMYK.pdf]

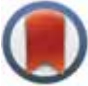

CrossMark

[click for updates](#)

Supplement: Supplementary material [file rsos191074supp1.zip › RS_crossmark_logo.pdf]

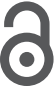

Supplement: Supplementary material [file rsos191074supp1.zip › openaccesslogo_bw.pdf]

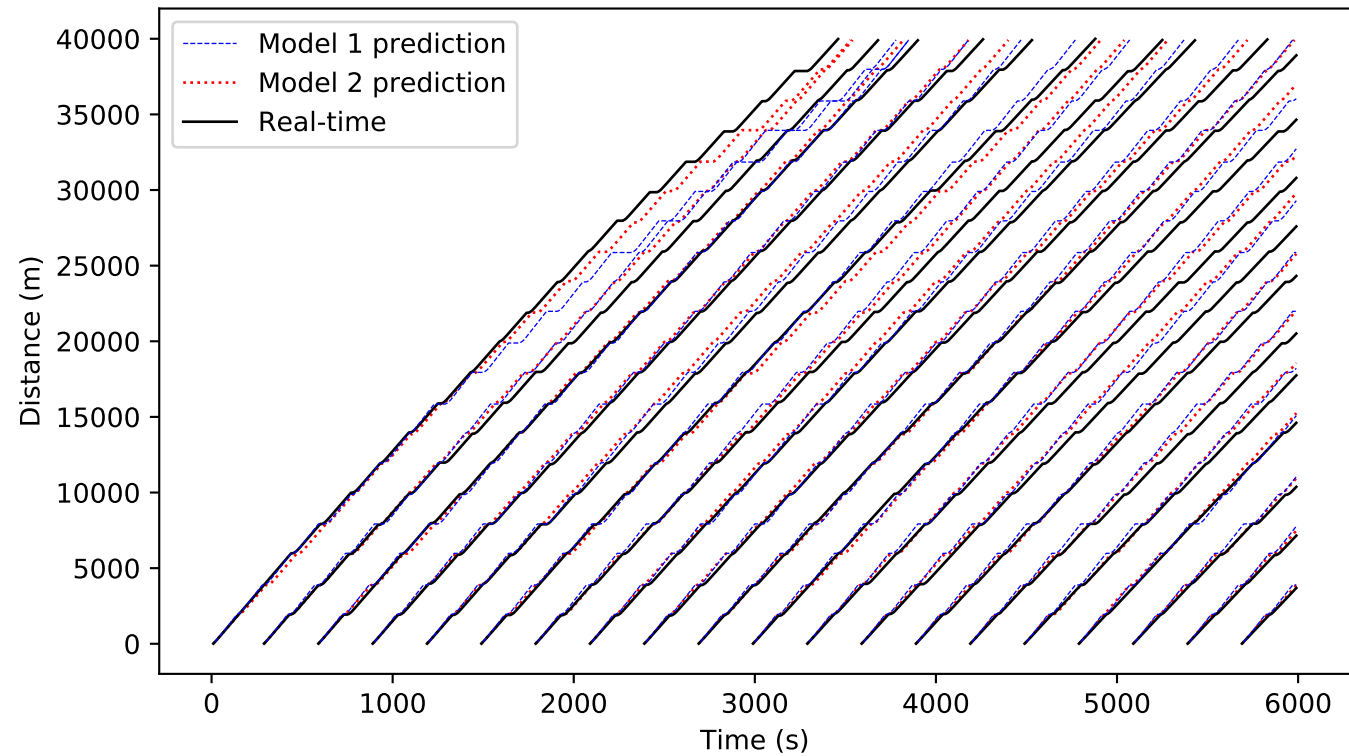

Supplement: Supplementary material [file rsos191074supp1.zip › Figures/Fig_calibration.pdf]

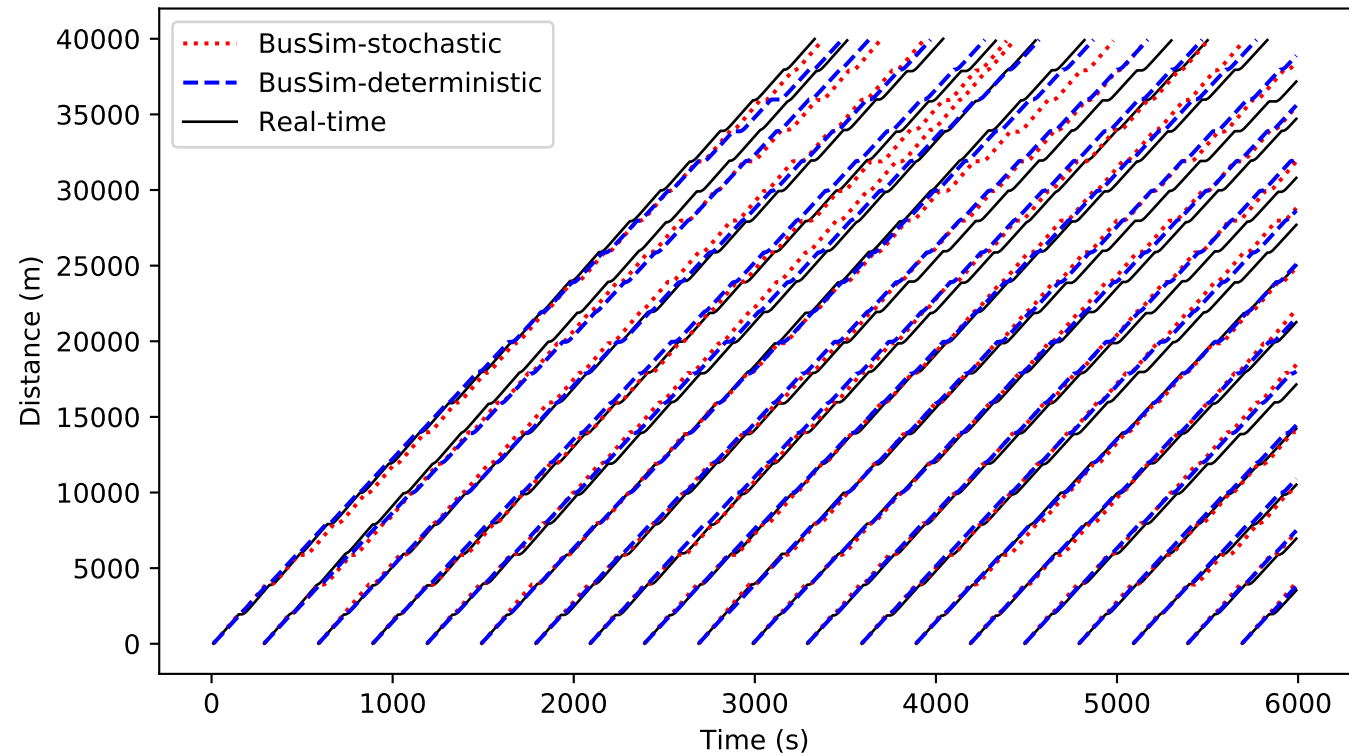

Supplement: Supplementary material [file rsos191074supp1.zip › Figures/Fig_calibration_IncreaseRate_7.pdf]

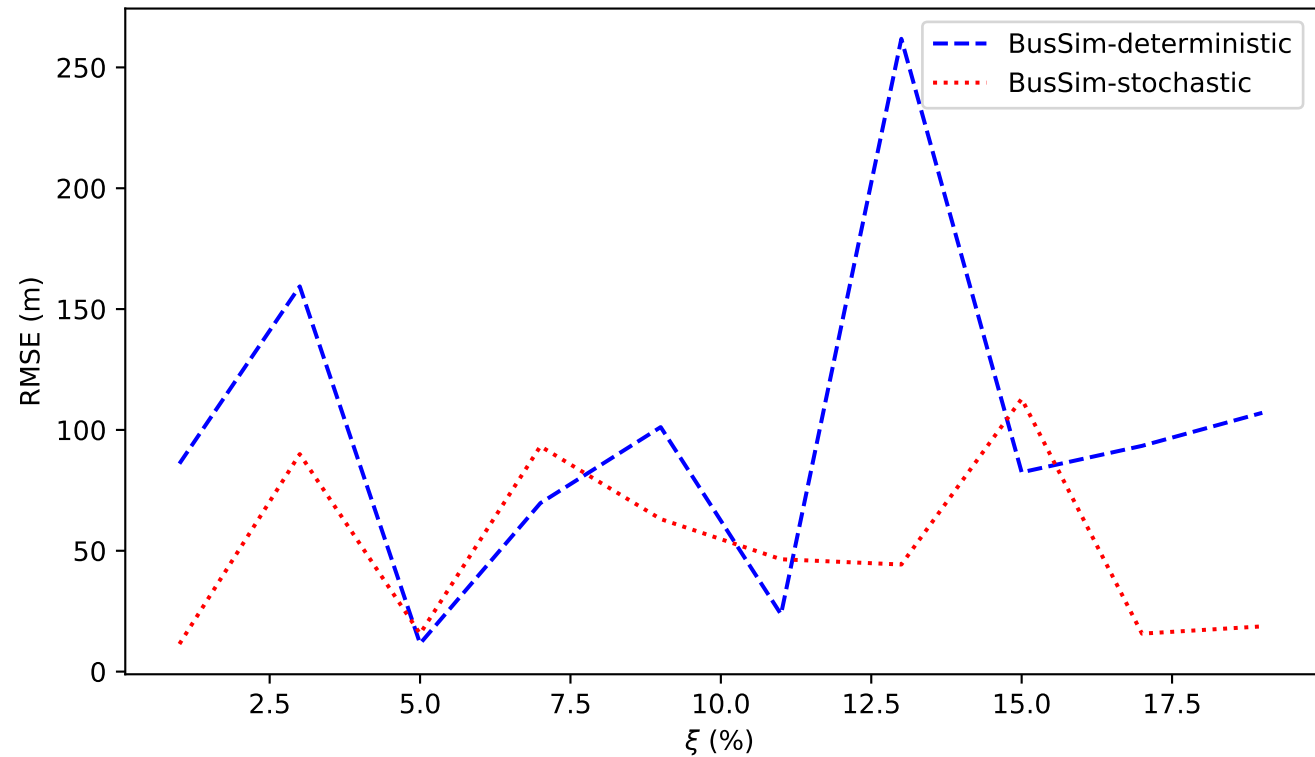

Supplement: Supplementary material [file rsos191074supp1.zip › Figures/Fig_calibration_results_IncreaseRate.pdf]

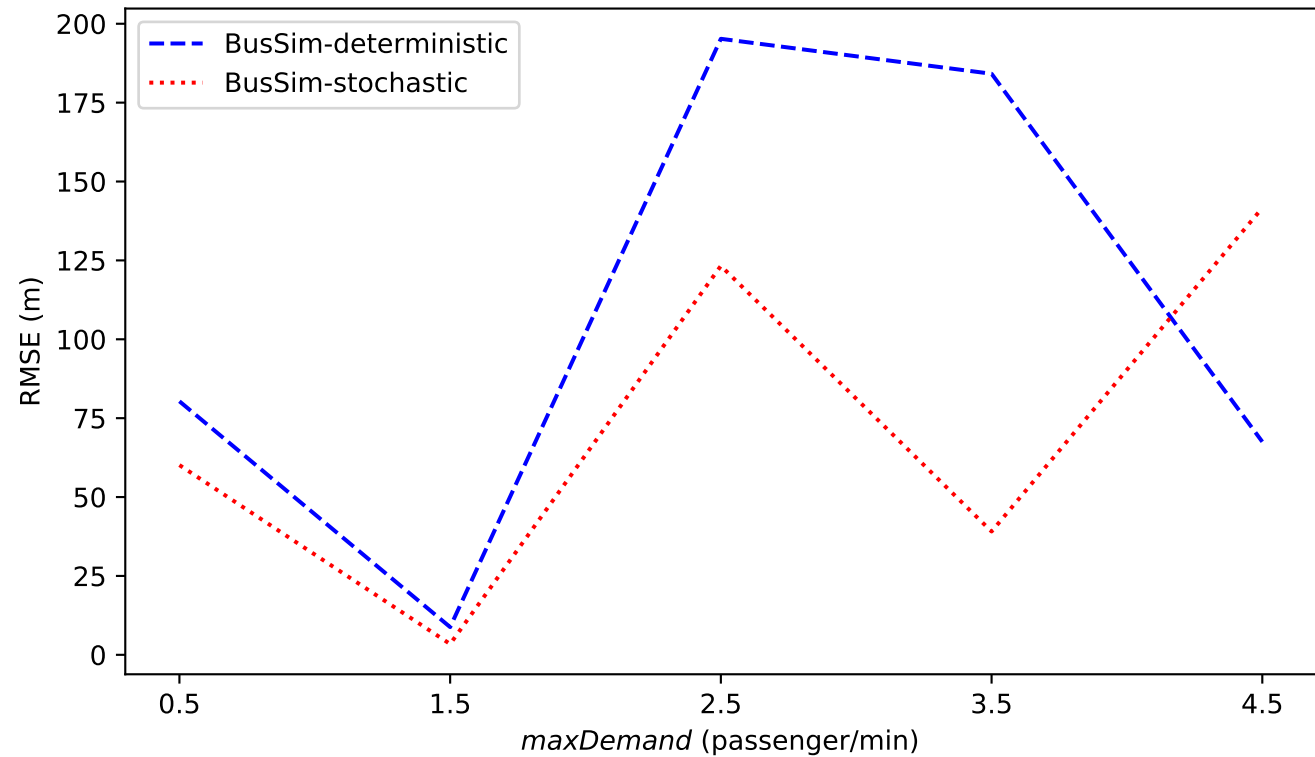

Supplement: Supplementary material [file rsos191074supp1.zip › Figures/Fig_calibration_results_maxDemand.pdf]

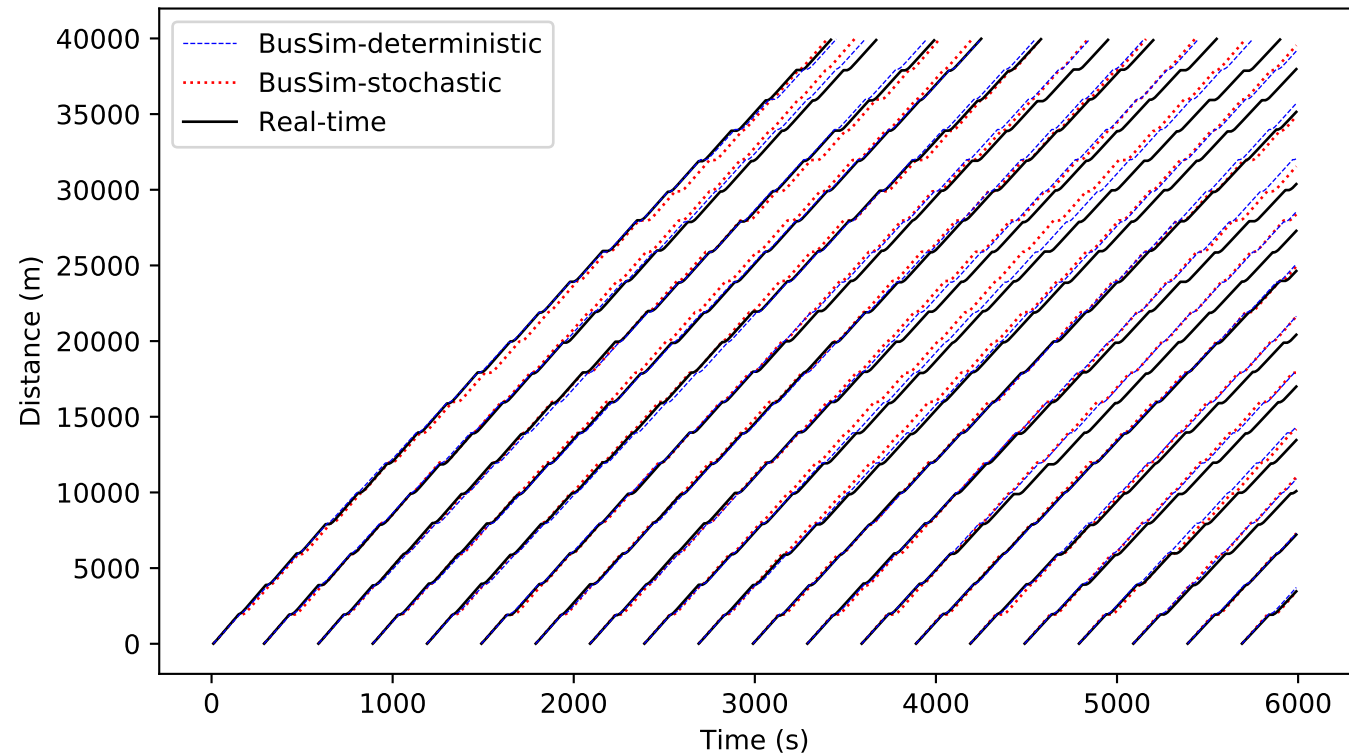

Supplement: Supplementary material [file rsos191074supp1.zip › Figures/Fig_calibration_v2.pdf]

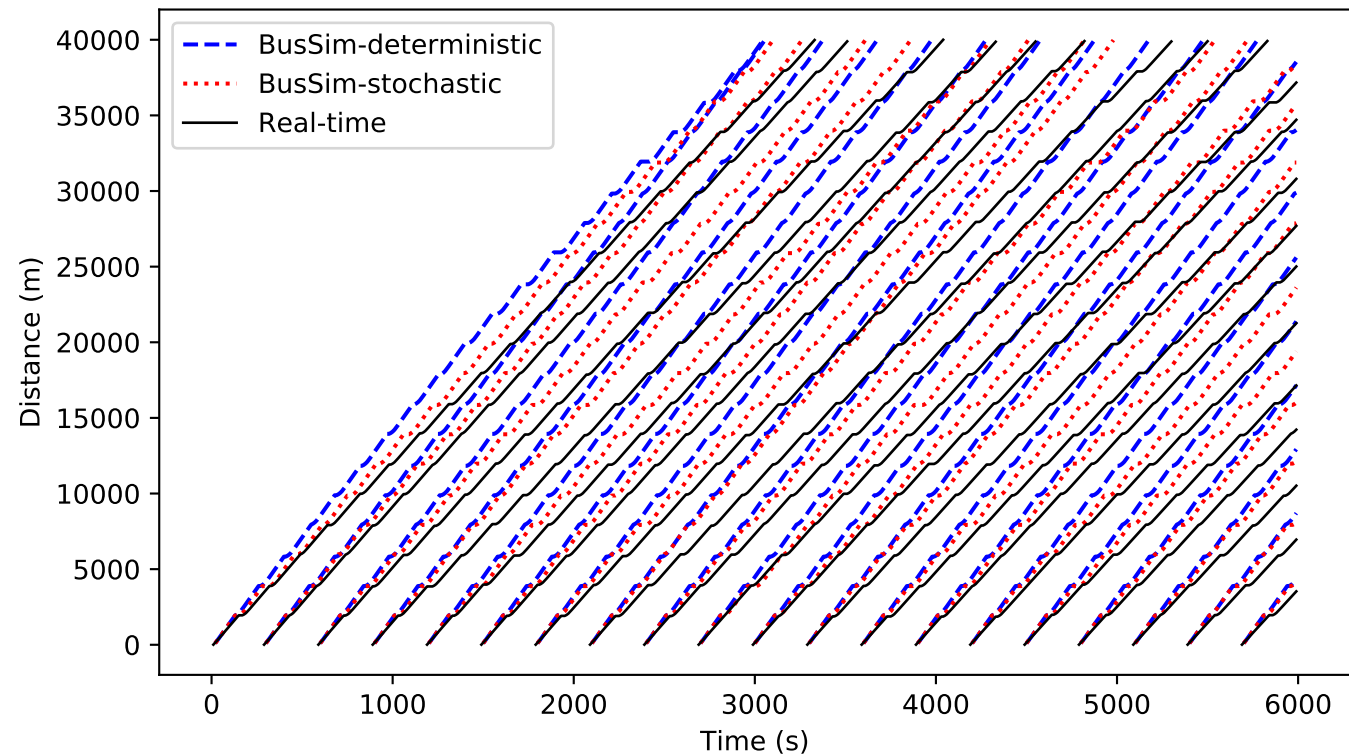

Supplement: Supplementary material [file rsos191074supp1.zip › Figures/Fig_do_nothing_IncreaseRate_7.pdf]

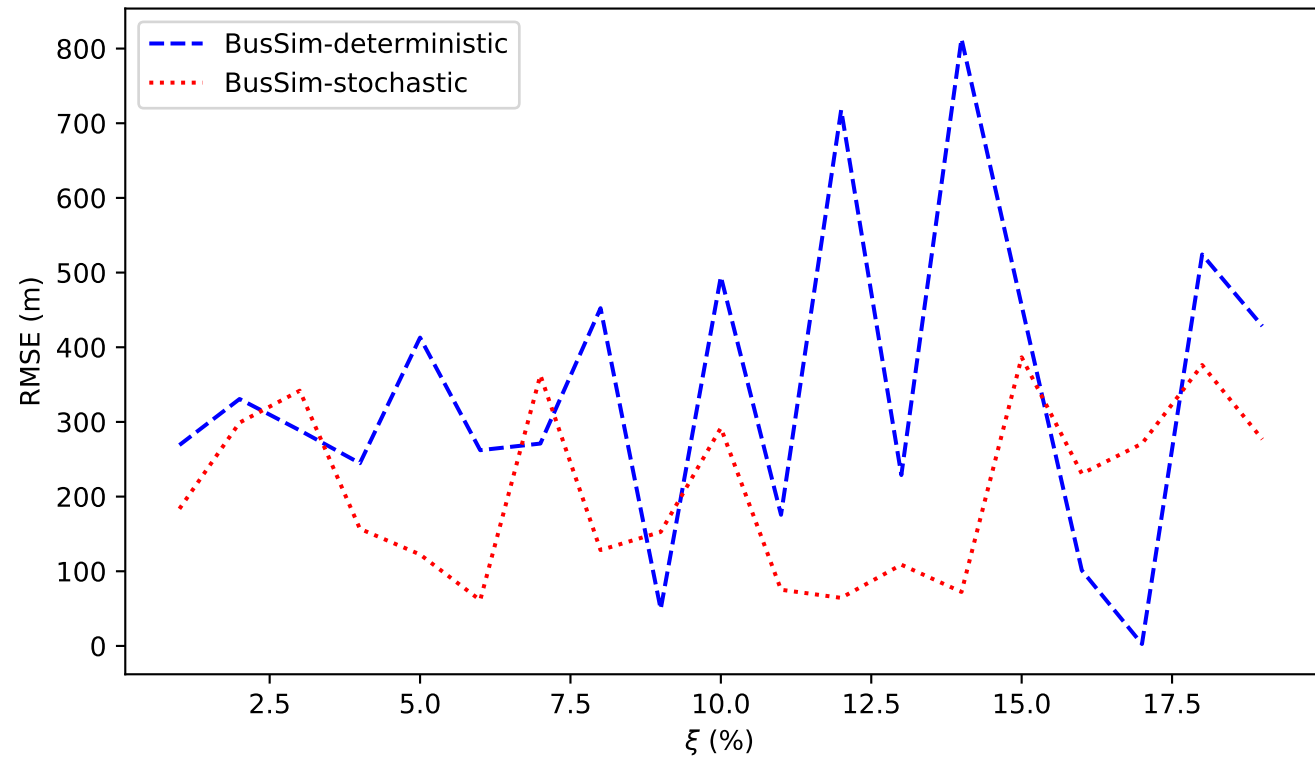

Supplement: Supplementary material [file rsos191074supp1.zip › Figures/Fig_do_nothing_results.pdf]

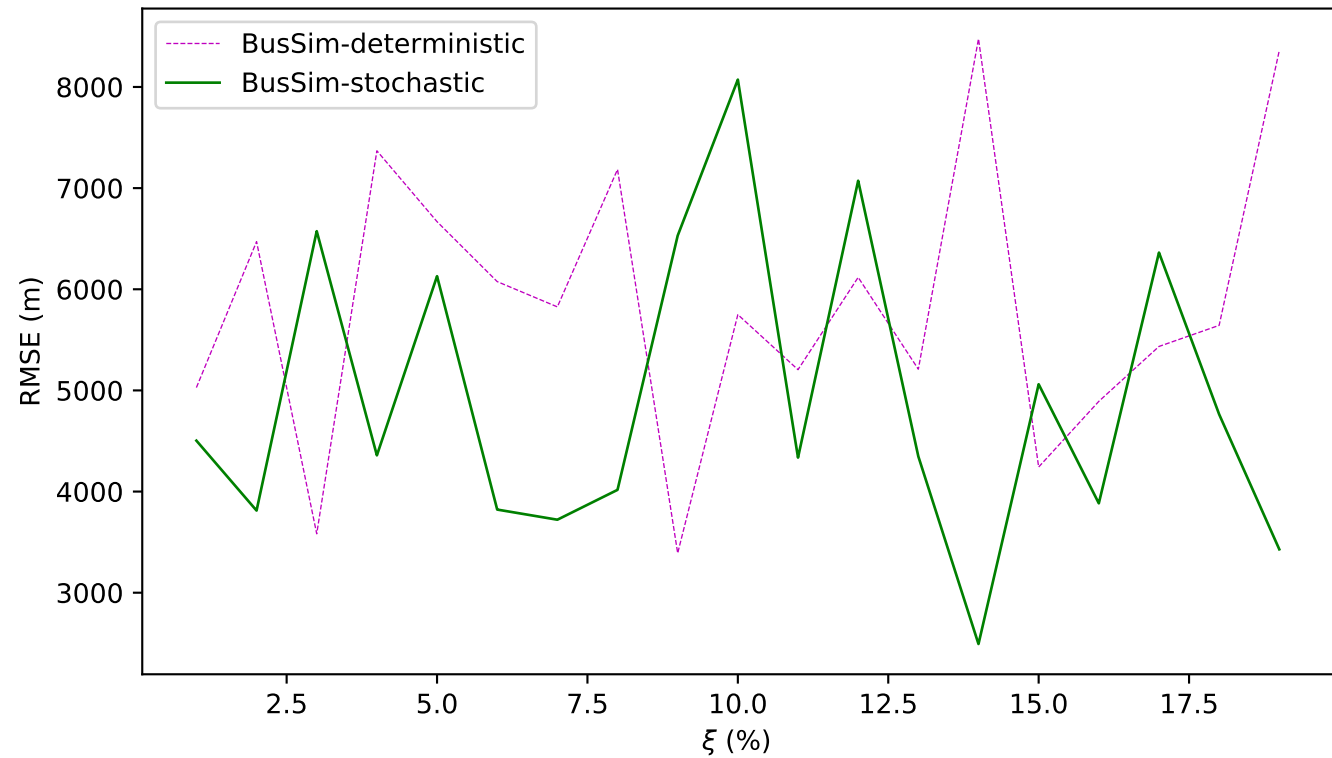

Supplement: Supplementary material [file rsos191074supp1.zip › Figures/Fig_do_nothing_results_IncreaseRate.pdf]

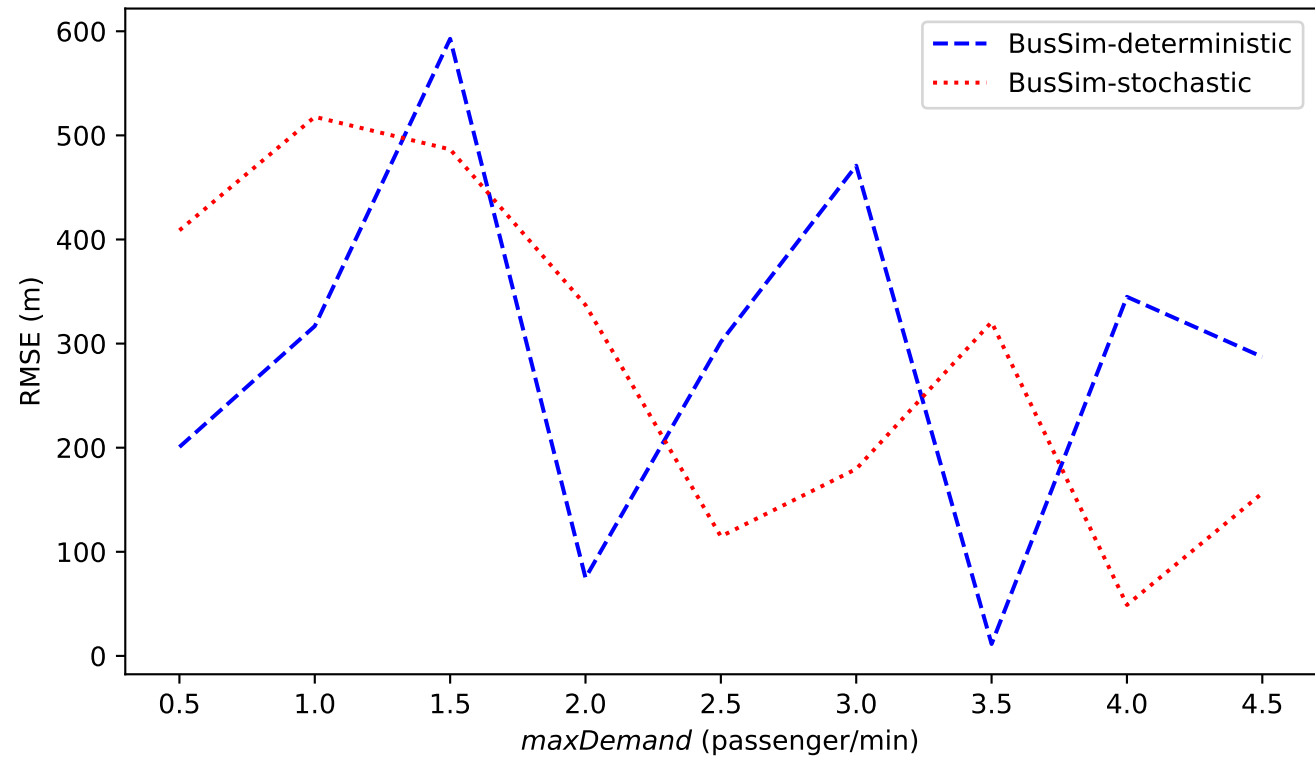

Supplement: Supplementary material [file rsos191074supp1.zip › Figures/Fig_do_nothing_results_maxDemand.pdf]

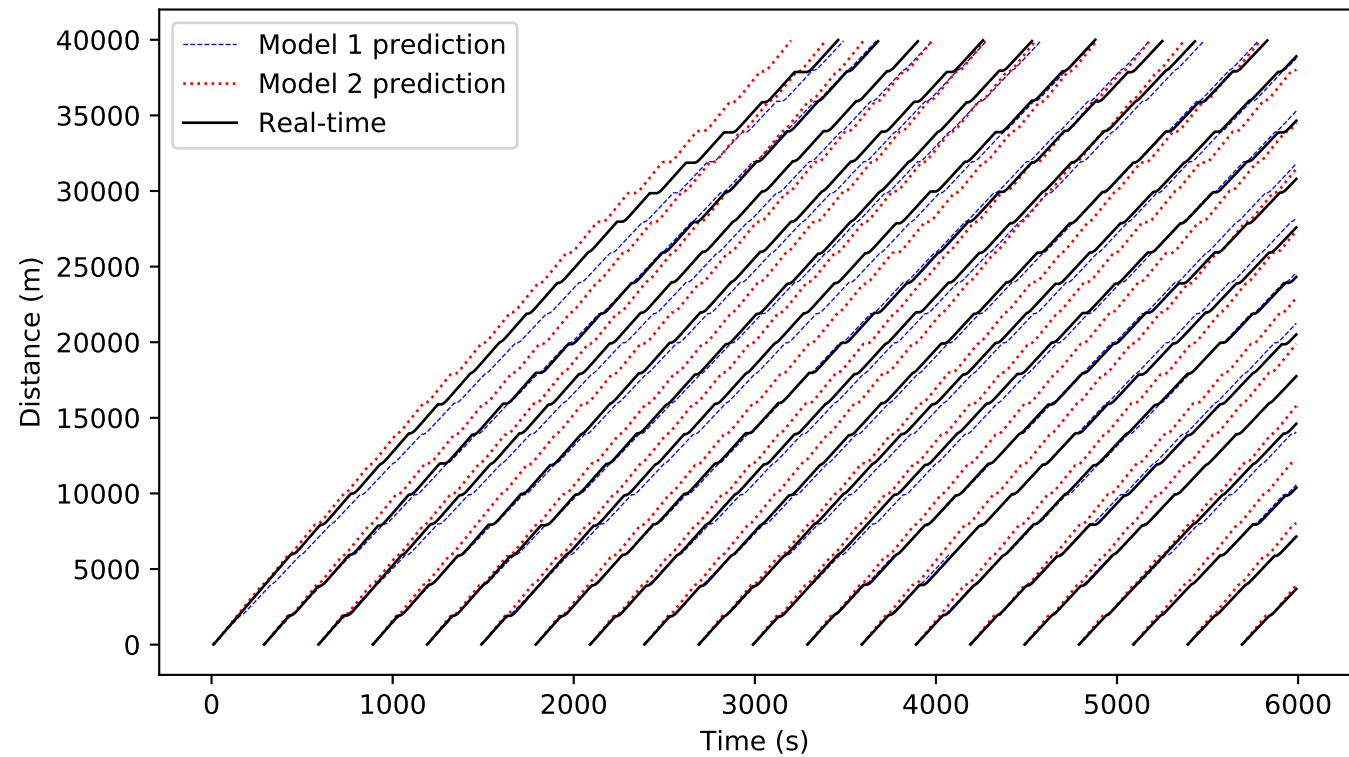

Supplement: Supplementary material [file rsos191074supp1.zip › Figures/Fig_do_nothing_static.pdf]

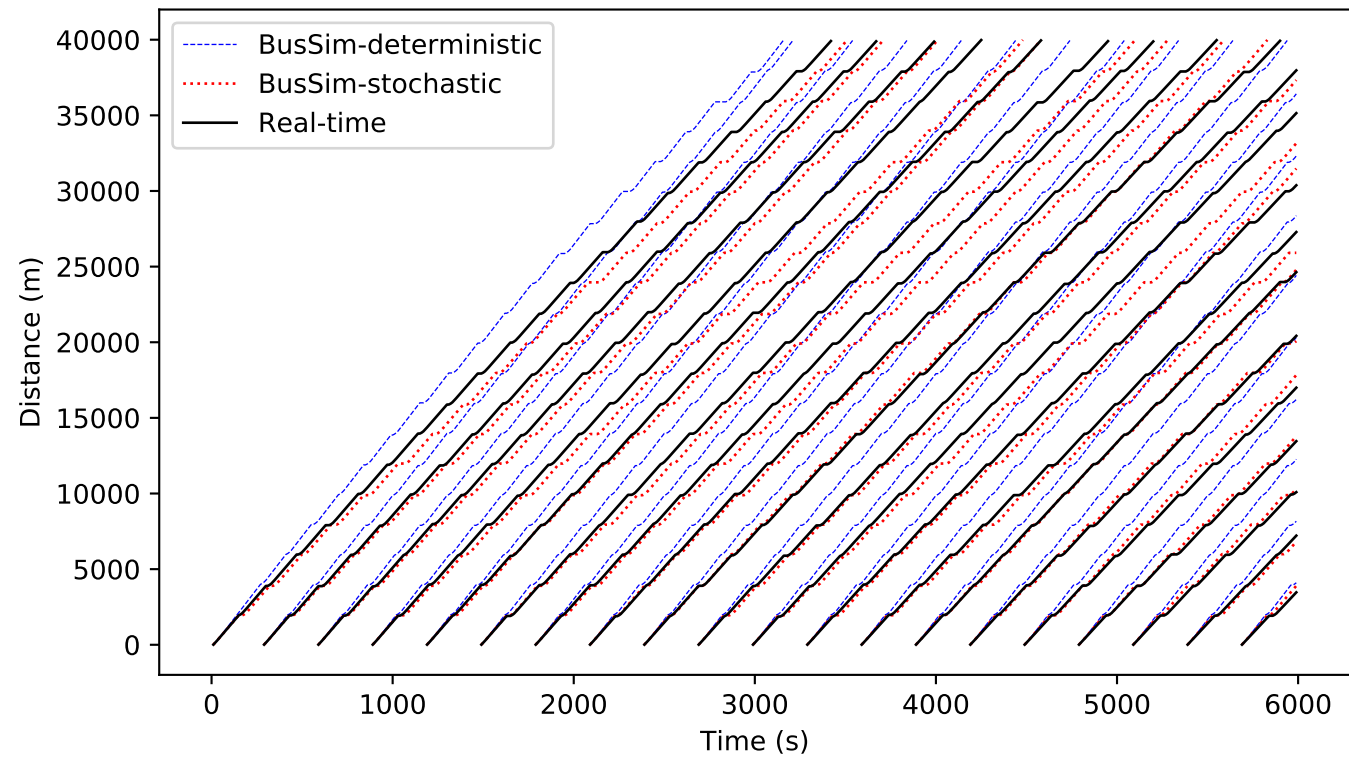

Supplement: Supplementary material [file rsos191074supp1.zip › Figures/Fig_do_nothing_static_v2.pdf]

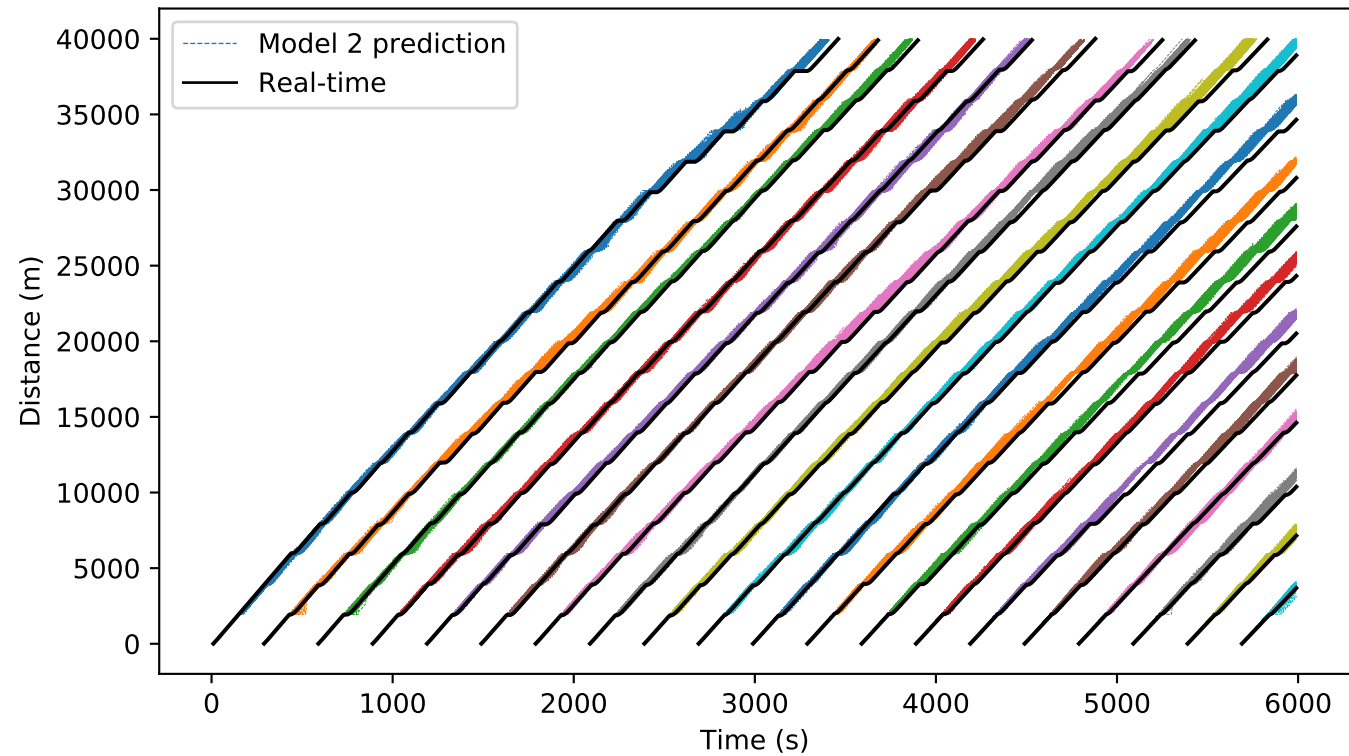

Supplement: Supplementary material [file rsos191074supp1.zip › Figures/Fig_PF.pdf]

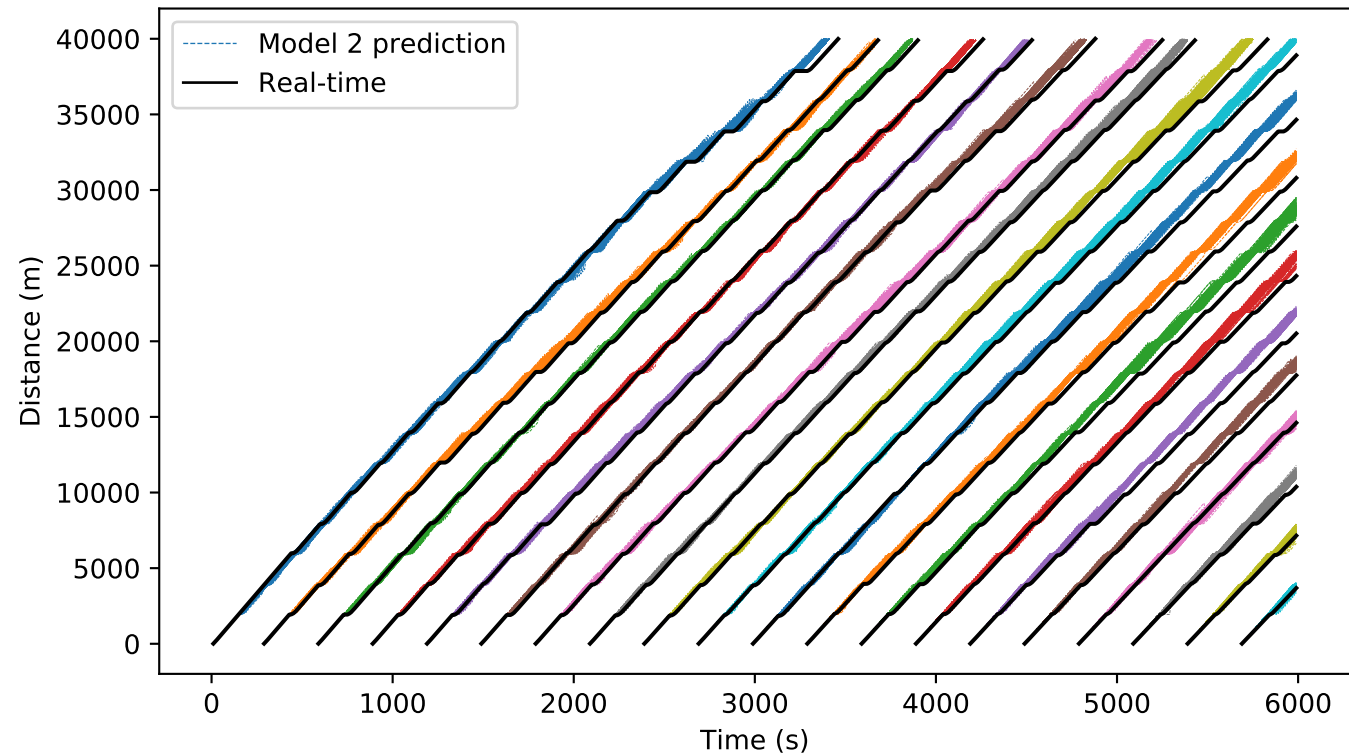

Supplement: Supplementary material [file rsos191074supp1.zip › Figures/Fig_PF_300.pdf]

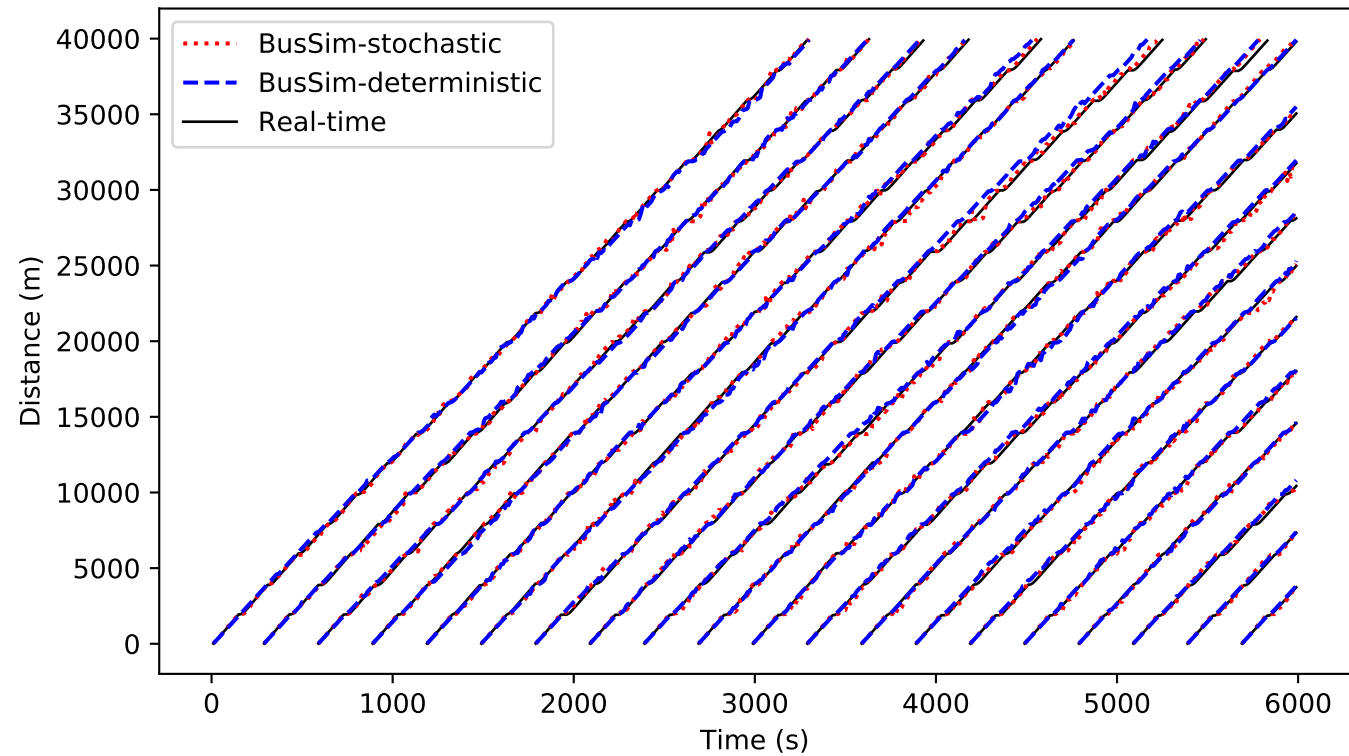

Supplement: Supplementary material [file rsos191074supp1.zip › Figures/Fig_PF_IncreaseRate_5.pdf]

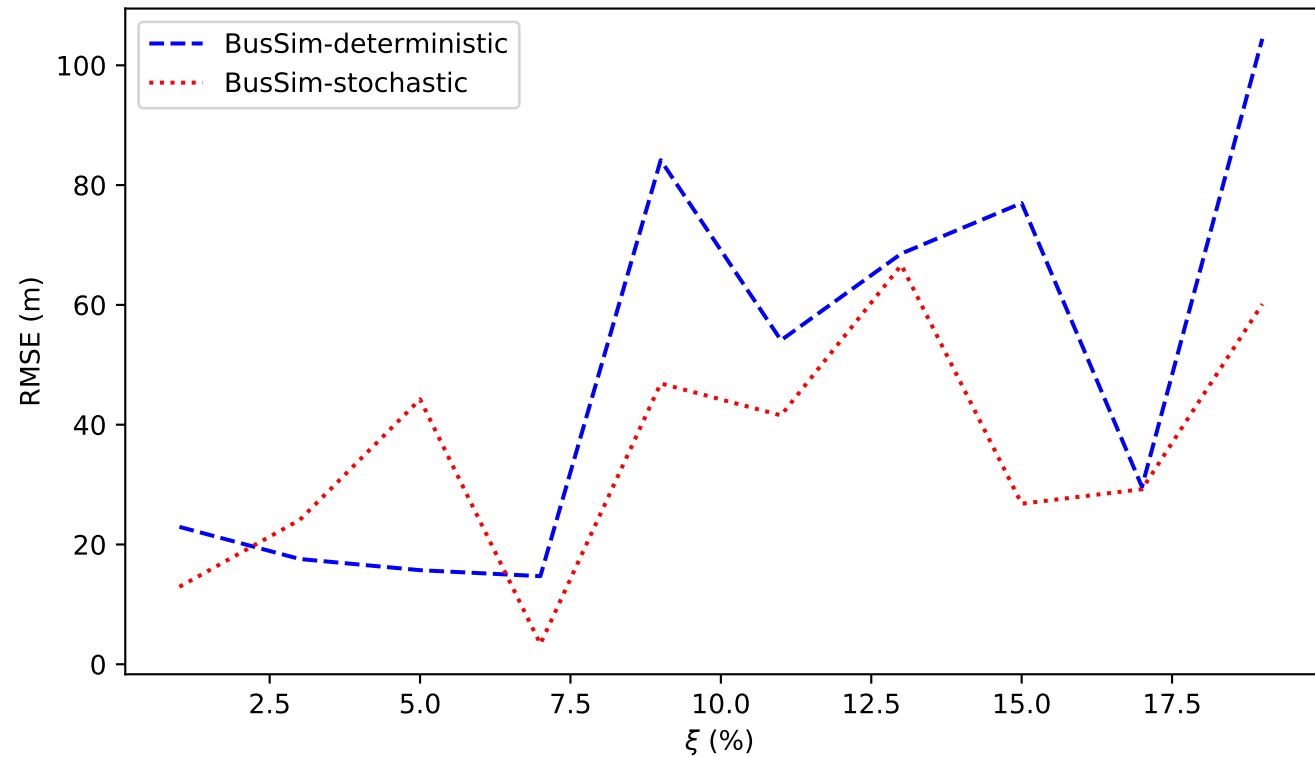

Supplement: Supplementary material [file rsos191074supp1.zip › Figures/Fig_PF_results_IncreaseRate.pdf]

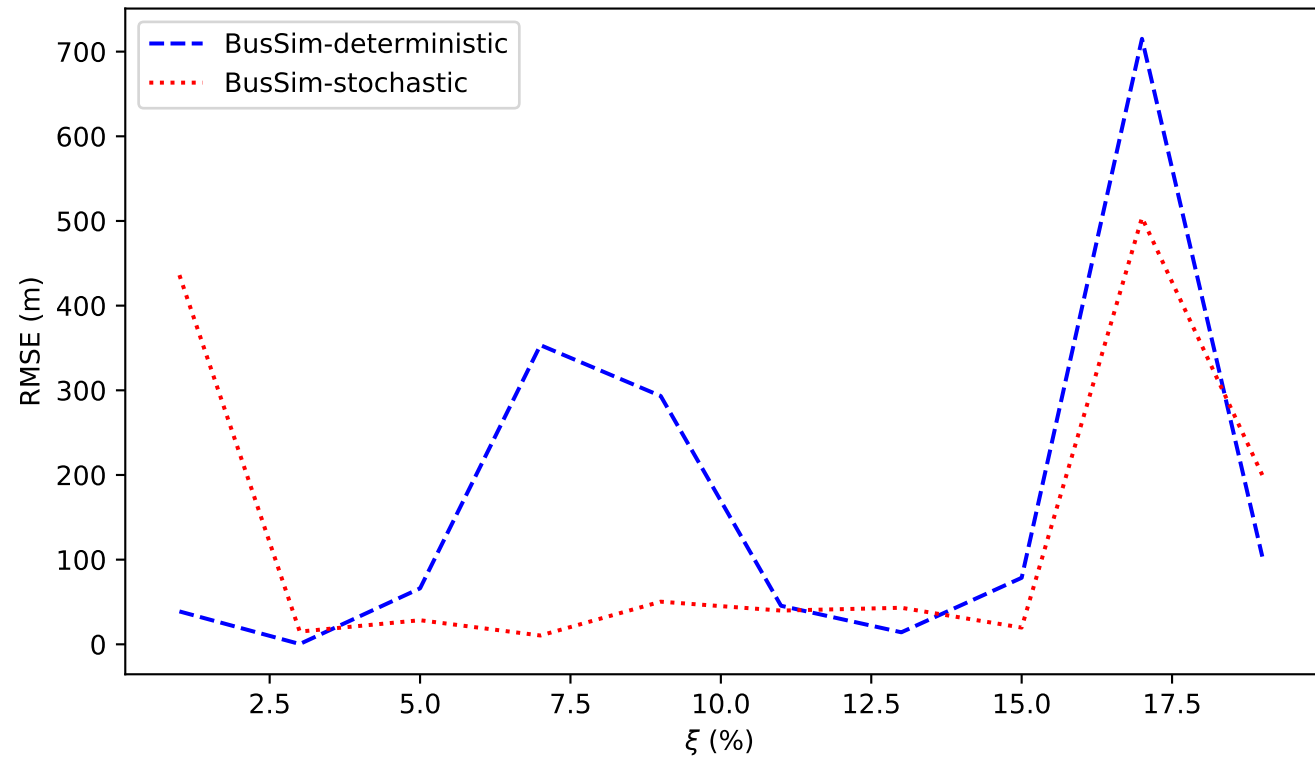

Supplement: Supplementary material [file rsos191074supp1.zip › Figures/Fig_PF_results_IncreaseRate_NO_CALIBRATION.pdf]

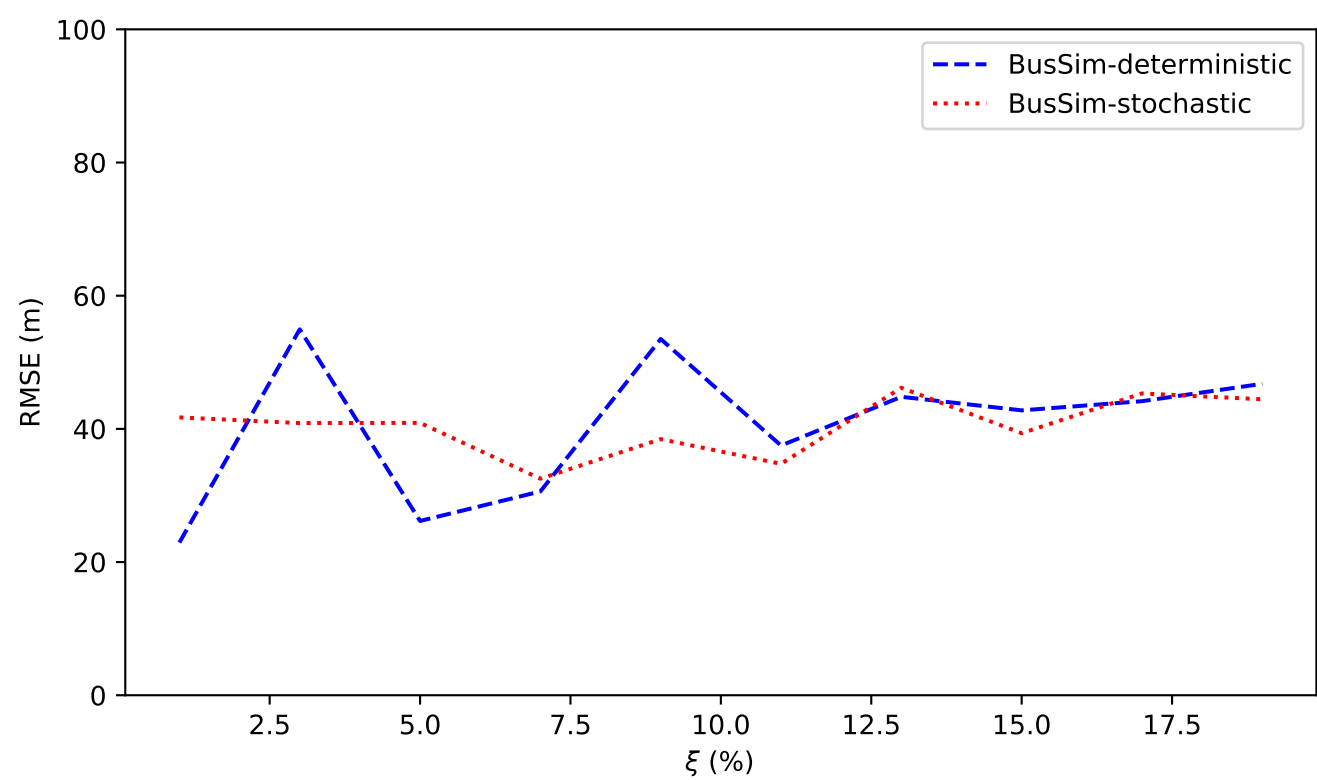

Supplement: Supplementary material [file rsos191074supp1.zip › Figures/Fig_PF_results_IncreaseRate_Replication.pdf]

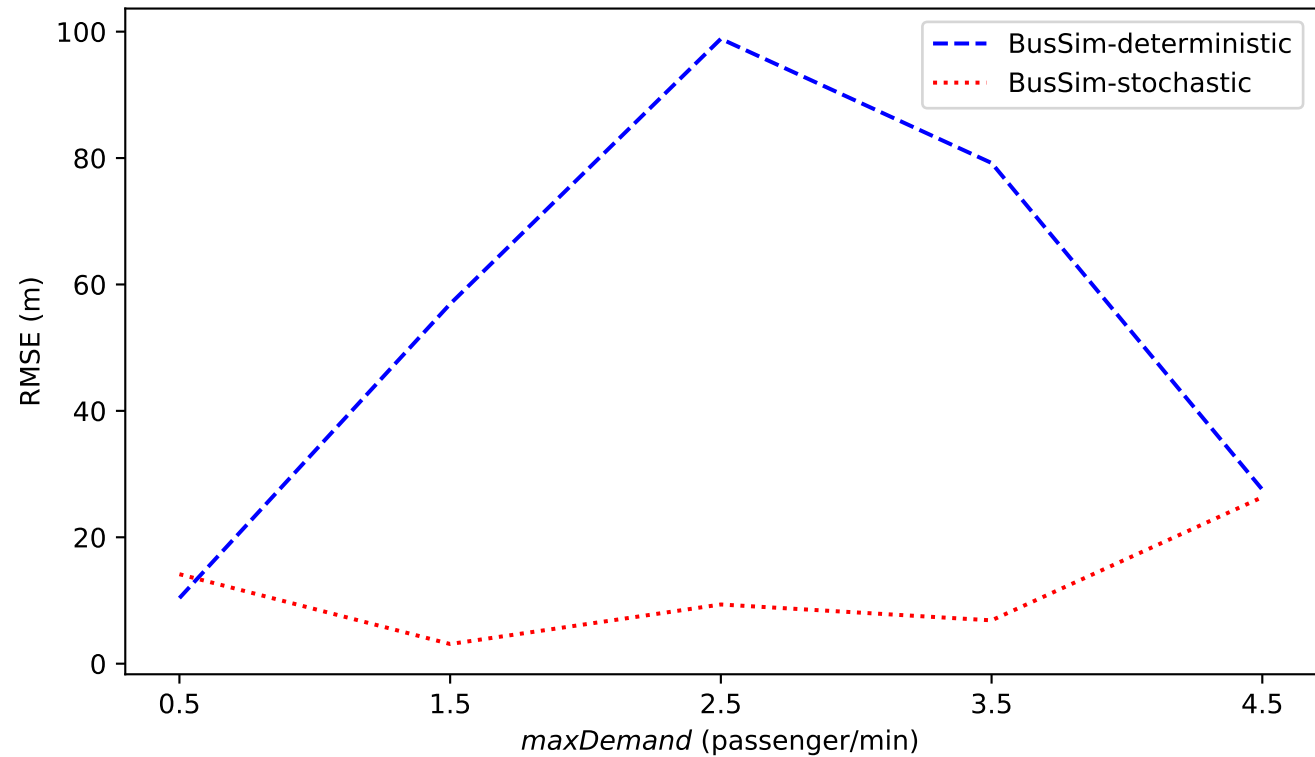

Supplement: Supplementary material [file rsos191074supp1.zip › Figures/Fig_PF_results_maxDemand.pdf]

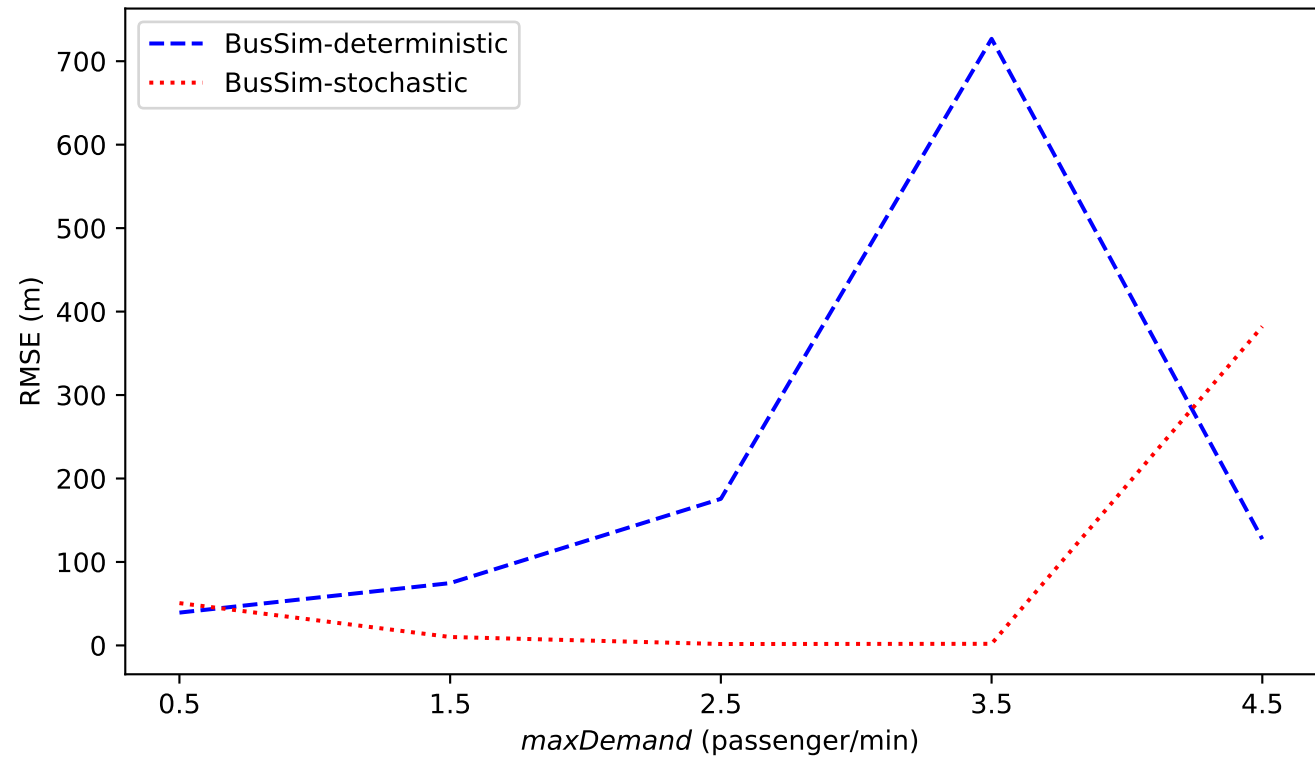

Supplement: Supplementary material [file rsos191074supp1.zip › Figures/Fig_PF_results_maxDemand_NO_CALIBRATION.pdf]

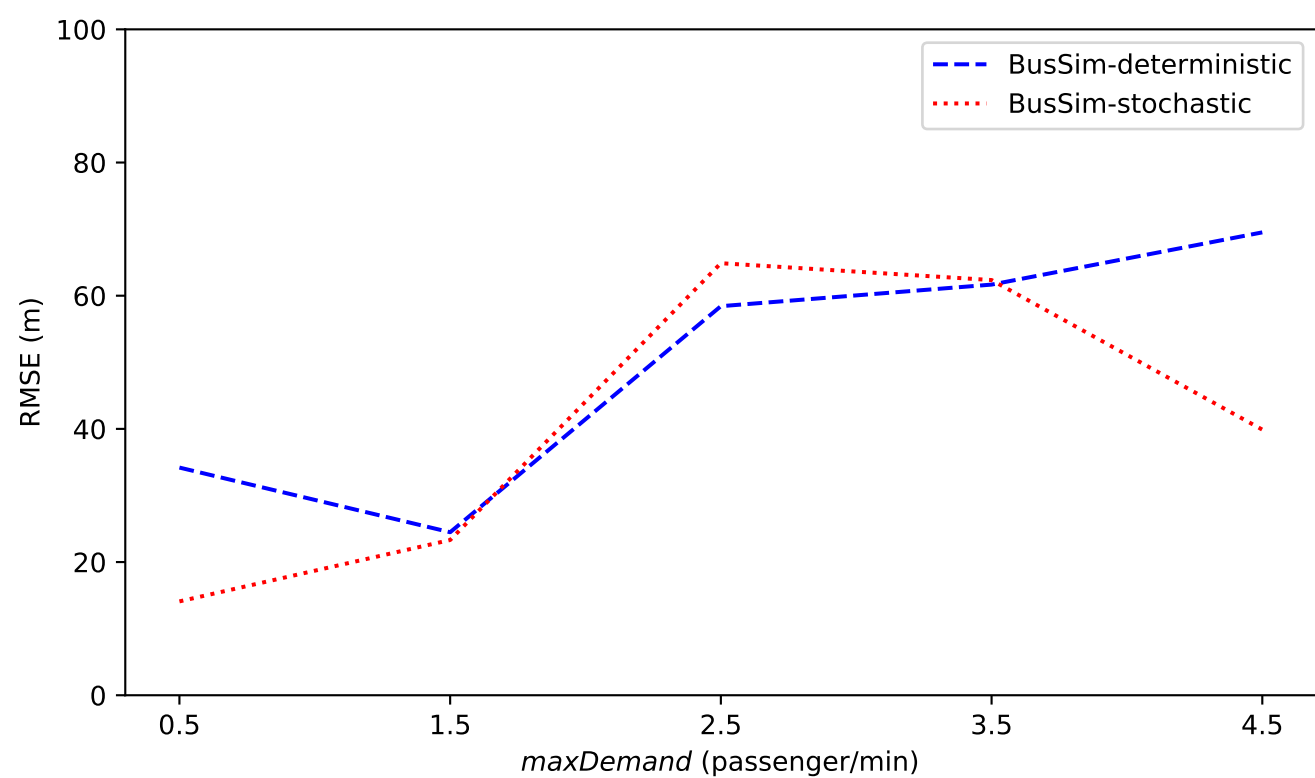

Supplement: Supplementary material [file rsos191074supp1.zip › Figures/Fig_PF_results_maxDemand_replication.pdf]

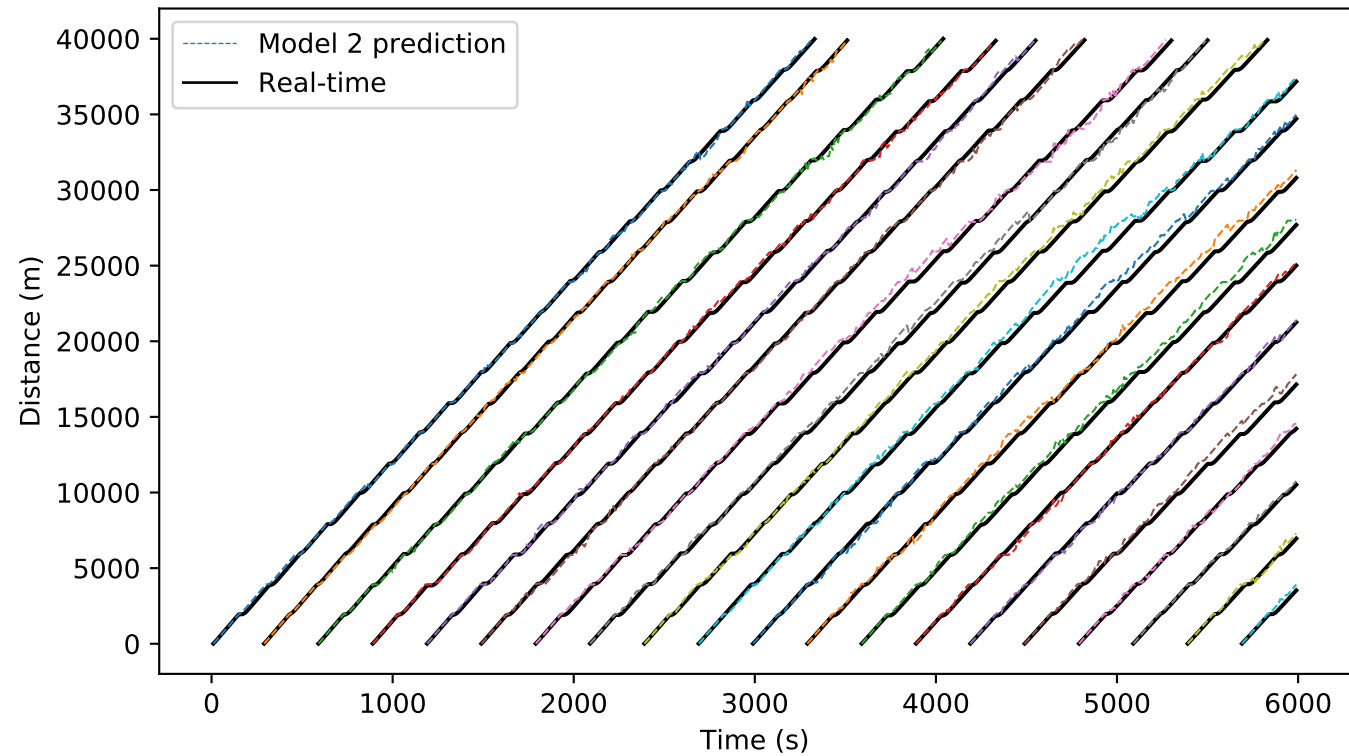

Supplement: Supplementary material [file rsos191074supp1.zip › Figures/Fig_PF_std00005_bestparticle.pdf]

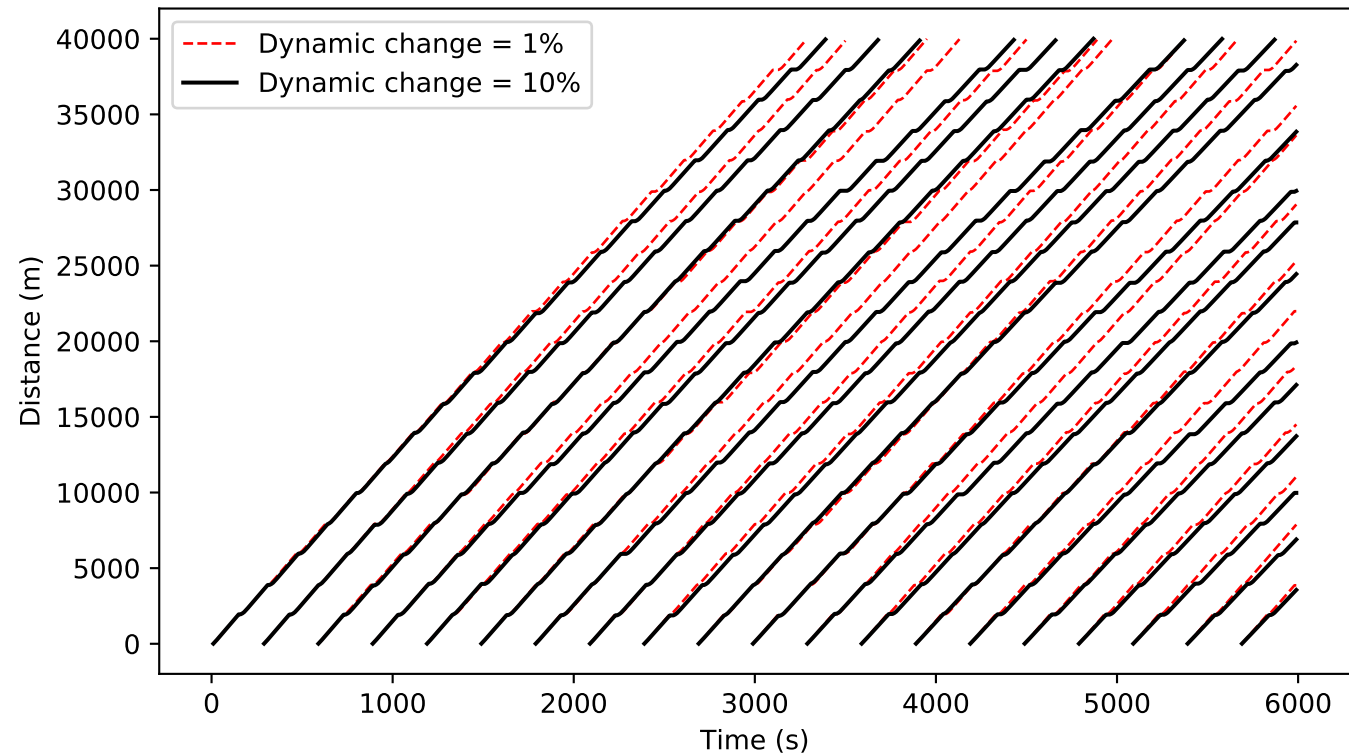

Supplement: Supplementary material [file rsos191074supp1.zip › Figures/Fig_spacetime_2dynamic.pdf]

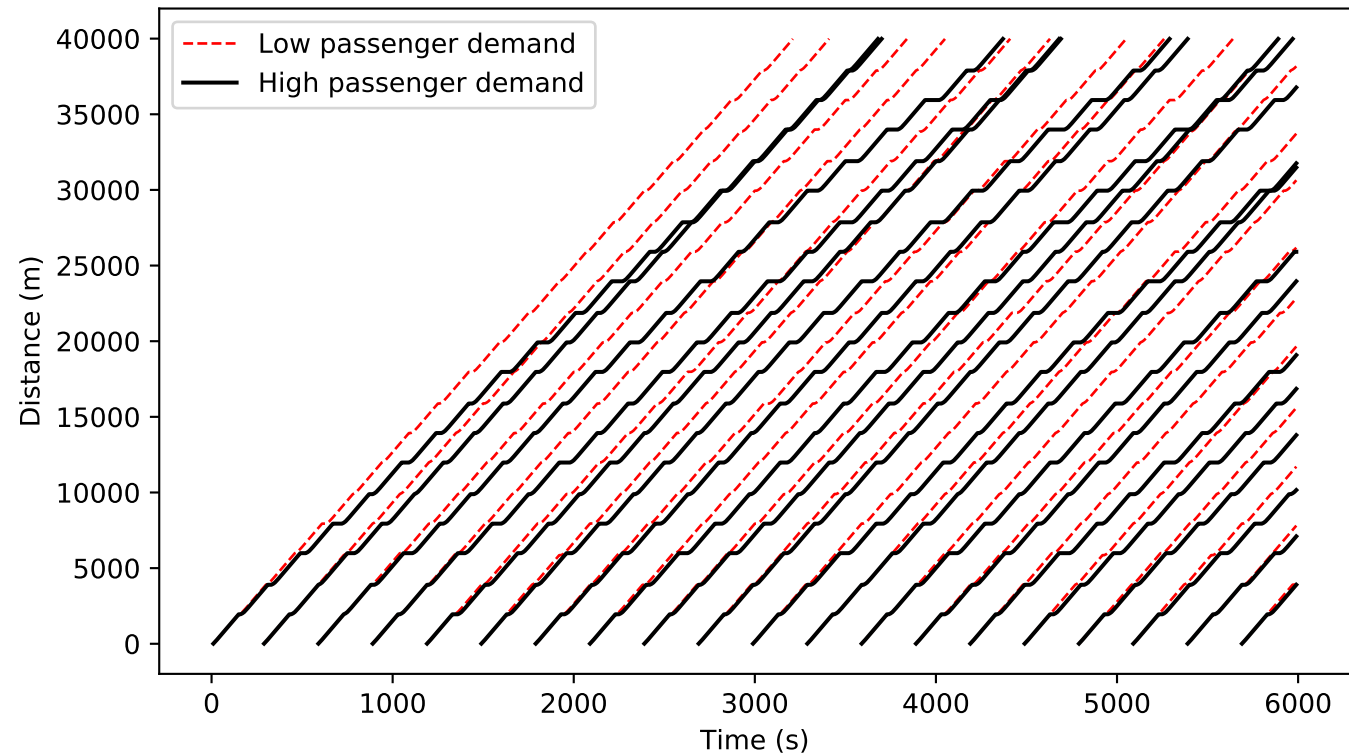

Supplement: Supplementary material [file rsos191074supp1.zip › Figures/Fig_spacetime_2stochastic.pdf]

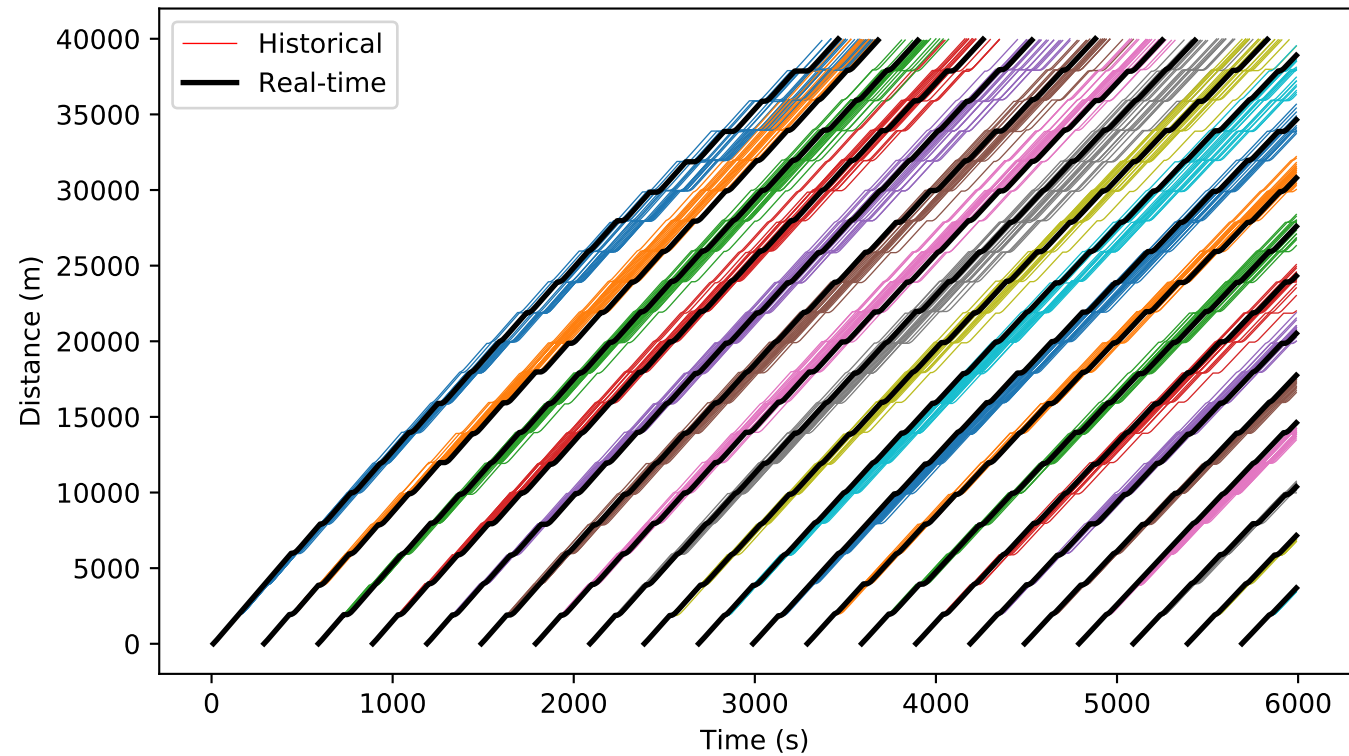

Supplement: Supplementary material [file rsos191074supp1.zip › Figures/Fig_spacetime_dynamic.pdf]

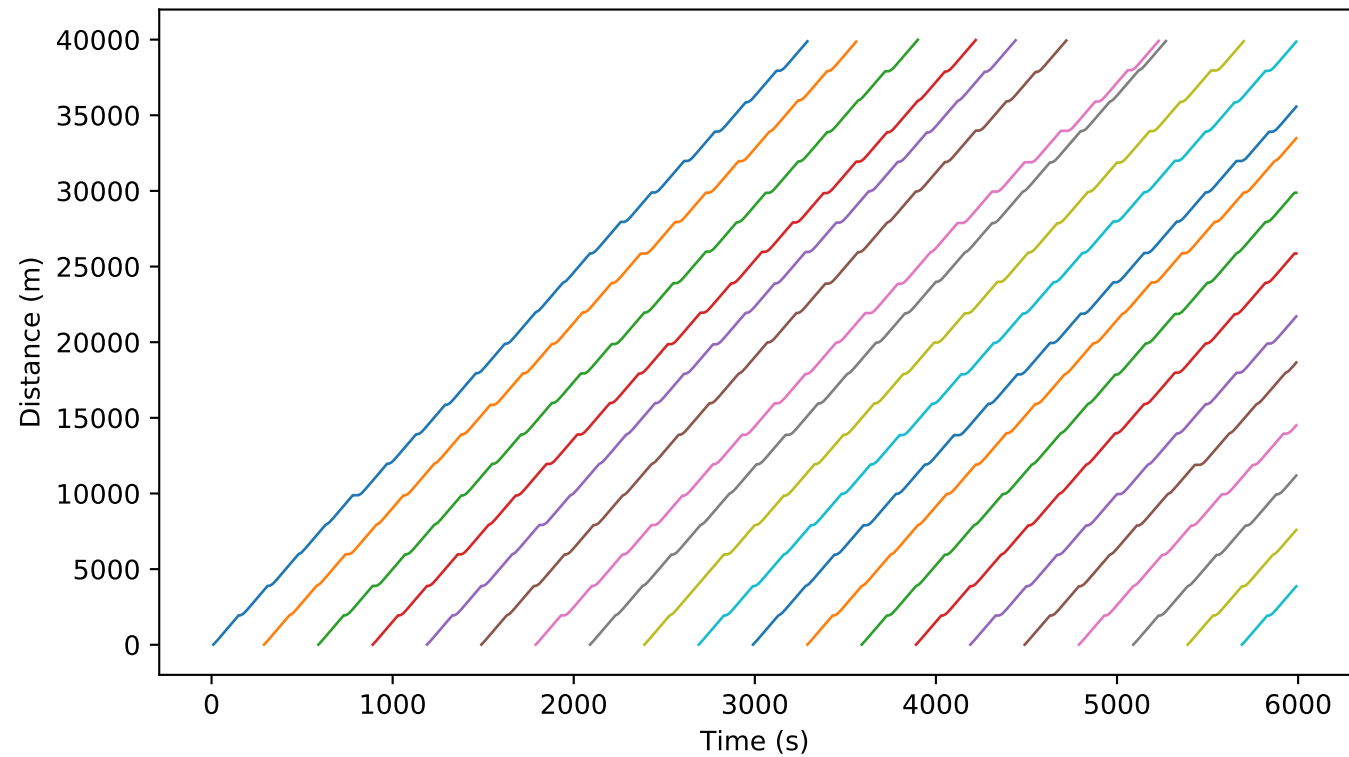

Supplement: Supplementary material [file rsos191074supp1.zip › Figures/Fig_spacetime_IncreaseRate_1.pdf]

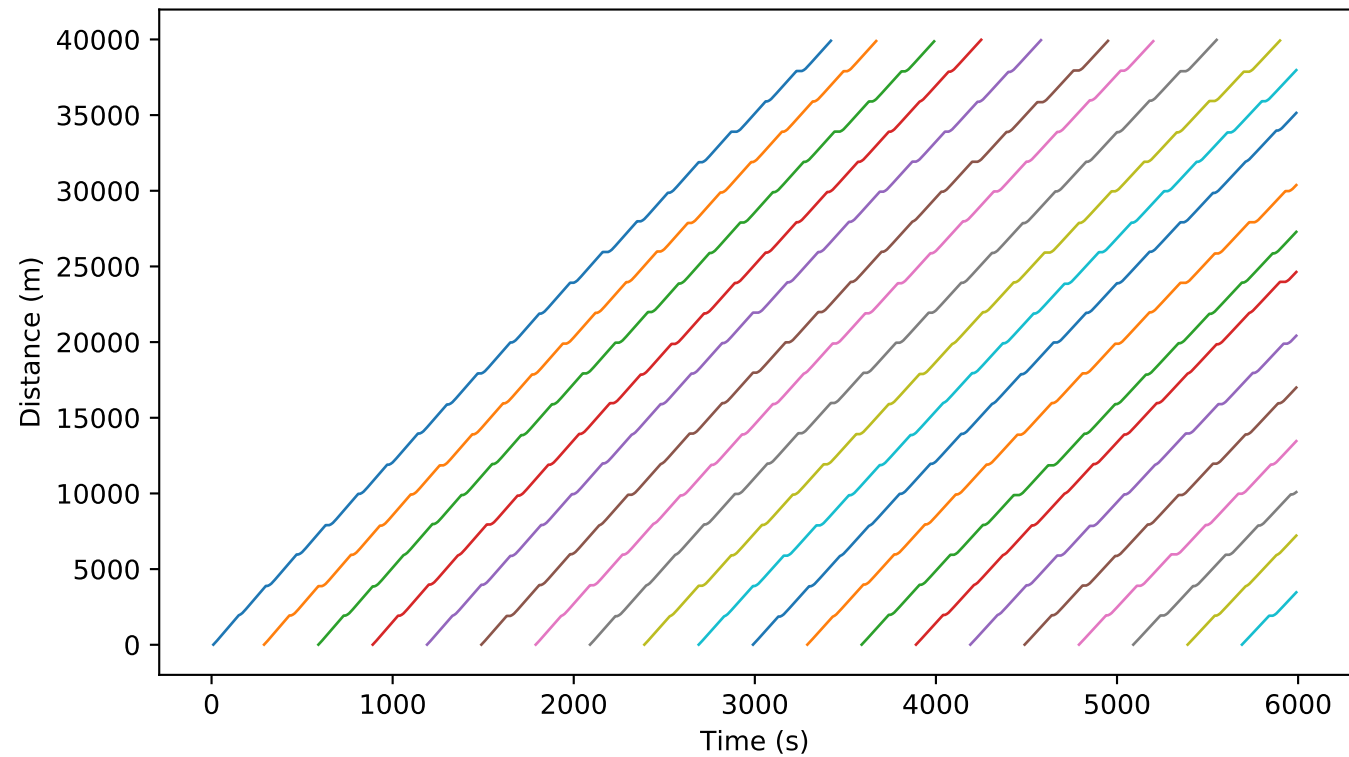

Supplement: Supplementary material [file rsos191074supp1.zip › Figures/Fig_spacetime_IncreaseRate_9.pdf]

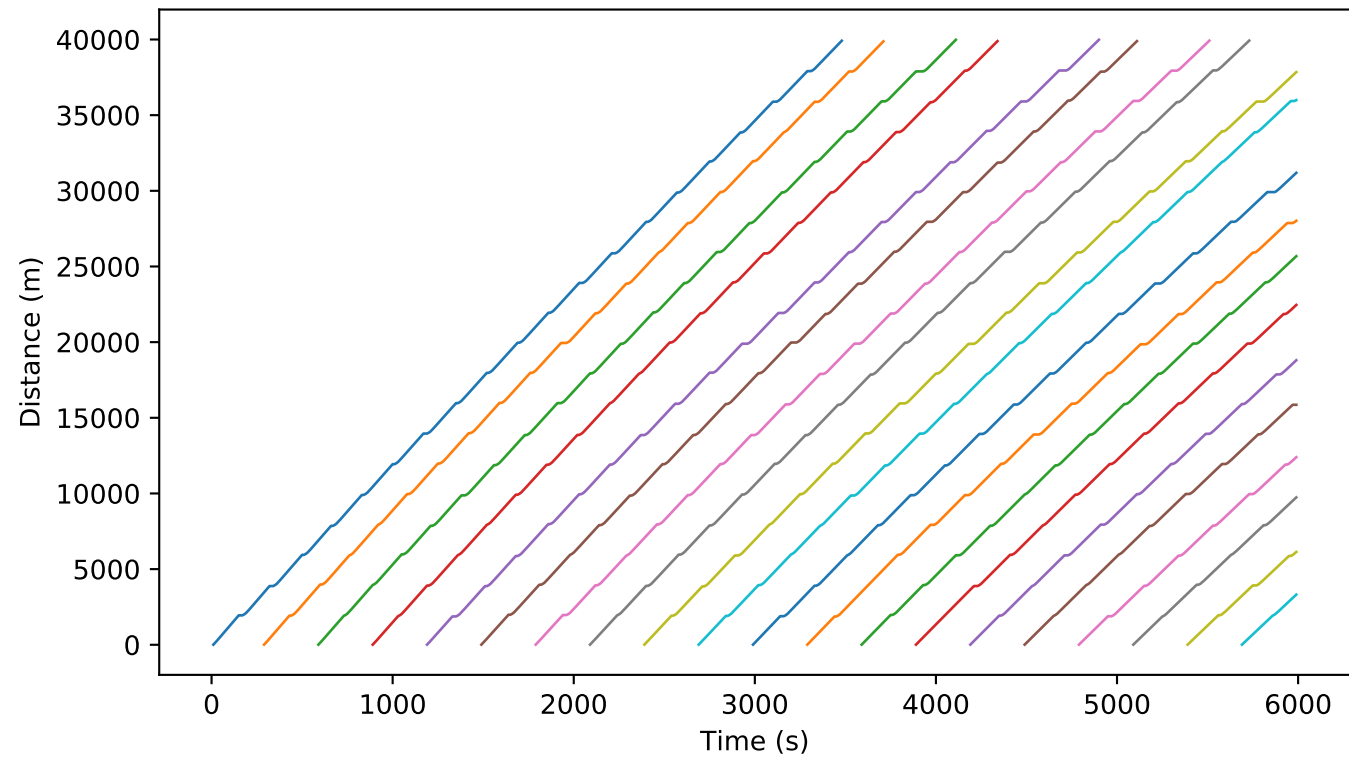

Supplement: Supplementary material [file rsos191074supp1.zip › Figures/Fig_spacetime_IncreaseRate_20.pdf]

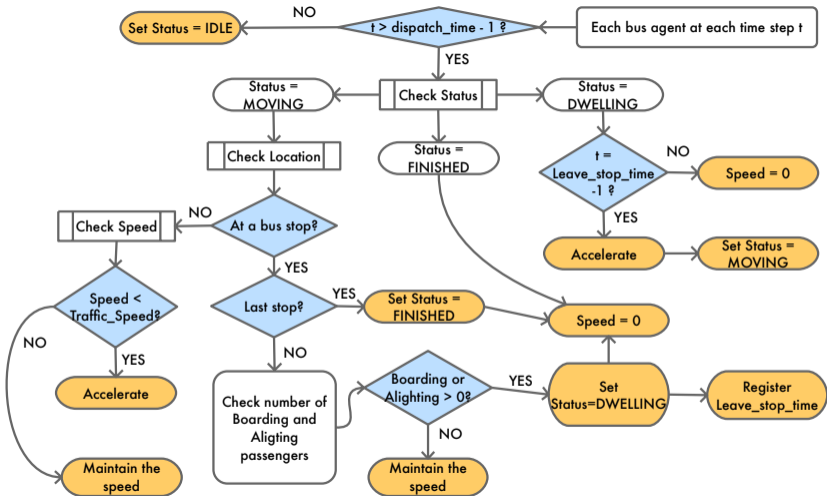

Supplement: Supplementary material [file rsos191074supp1.zip › Figures/flowchart.pdf]

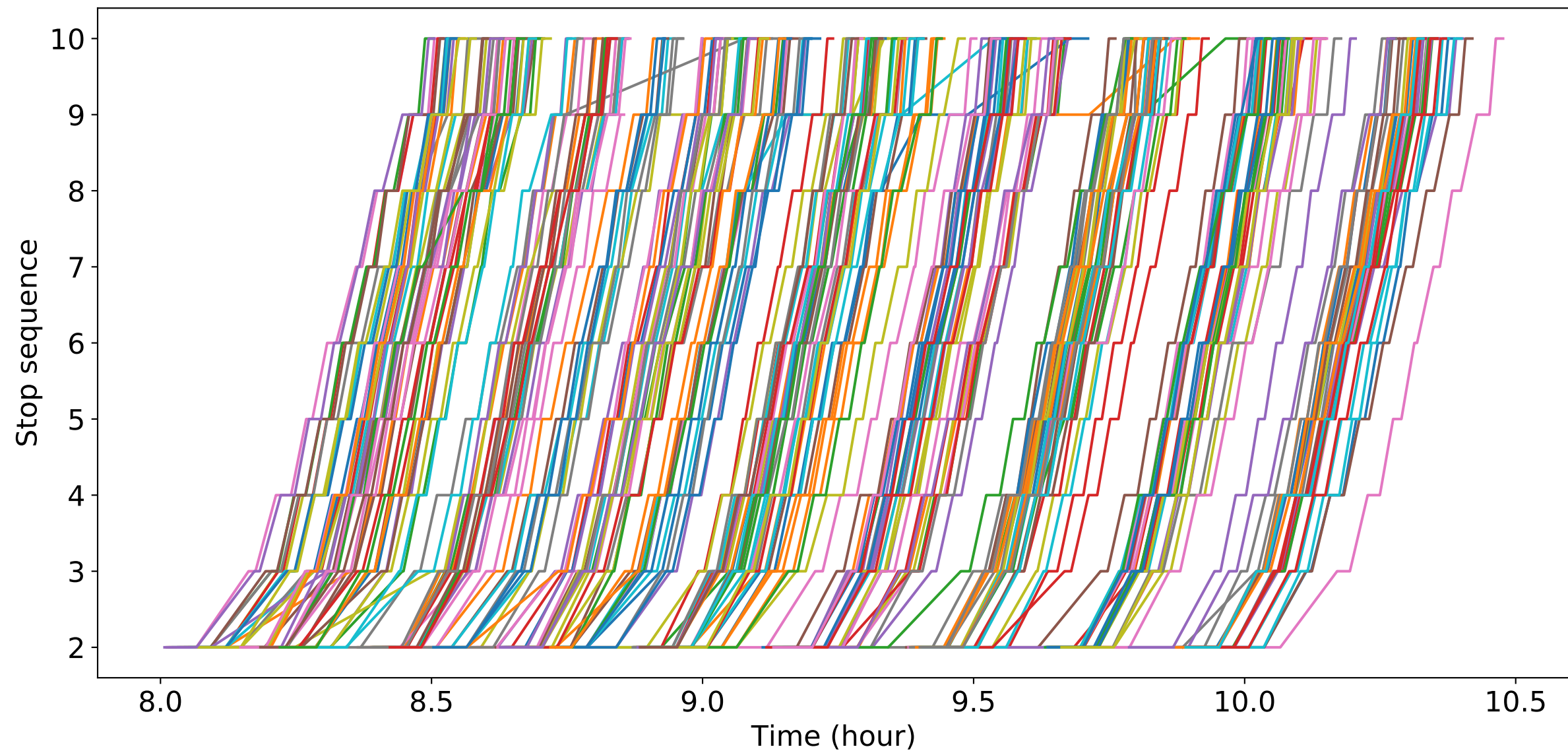

Supplement: Supplementary material [file rsos191074supp1.zip › Figures/AVL_Route555_2015.pdf]
